# Functional role of the biofilm regulator CsgD in *Salmonella enterica* sv. Typhi

**DOI:** 10.3389/fcimb.2024.1478488

**Published:** 2024-12-11

**Authors:** Juan F. González, Baileigh Laipply, Victoria A. Sadowski, Matthew Price, John S. Gunn

**Affiliations:** ^1^ Center for Microbial Pathogenesis, Abigail Wexner Research Institute at Nationwide Children’s Hospital, Columbus, OH, United States; ^2^ Infectious Diseases Institute, The Ohio State University, Columbus, OH, United States; ^3^ Department of Pediatrics, College of Medicine, The Ohio State University, Columbus, OH, United States

**Keywords:** *Salmonella*, curli, biofilm, cellulose, typhoid

## Abstract

**Introduction:**

Typhoid fever is an infectious disease primarily caused by *Salmonella enterica* sv. Typhi (*S.* Typhi), a bacterium that causes as many as 20 million infections and 600,000 deaths annually. Asymptomatic chronic carriers of S. Typhi play a major role in the transmission of typhoid fever, as they intermittently shed the bacteria and can unknowingly infect humans in close proximity. An estimated 90% of chronic carriers have gallstones; biofilm formation on gallstones is a primary factor in the establishment and maintenance of gallbladder carriage. CsgD is a central biofilm regulator in *Salmonella*, but the *S.* Typhi *csgD* gene has a mutation that introduces an early stop codon, resulting in a protein truncated by 8 amino acids at the C-terminus. In this study, we investigate the role of role of CsgD in *S.* Typhi.

**Methods:**

We introduced a fully functional copy of the *csgD* gene from *S.* Typhimurium into *S.* Typhi under both a native and a constitutive promoter and tested for red, dry, and rough (Rdar) colony morphology, curli fimbriae, cellulose, and biofilm formation.

**Results and discussion:**

We demonstrate that although CsgD-regulated curli and cellulose production were partially restored, the introduction of the *S.* Typhimurium *csgD* did not induce the Rdar colony morphology. Interestingly, we show that CsgD does not have a significant role in S. Typhi biofilm formation, as biofilm-forming capacities depend more on the isolate than the CsgD regulator. This data suggests the presence of an alternative biofilm regulatory process in this human-restricted pathogen.

## Introduction

Typhoid Fever is a global human-specific illness caused primarily by *Salmonella enterica* serovar Typhi (*S.* Typhi). Approximately 5% of those infected with *S.* Typhi that resolve an acute infection become chronic carriers, with the gallbladder (GB) being the primary site of carriage. Because *S.* Typhi is a human specific pathogen, carriers are thought to be responsible for much of the human-to-human spread of disease, and in fact, there is no known or suspected non-human reservoir (Gonzalez-Escobedo and Gunn, 2013; Dougan and Baker, 2014). The conditions linked to *Salmonella* GB carriage, including the association with gallstones (GSs) (Schiøler et al., 1983), confinement to an organ, and recalcitrance to antibiotics or immune clearance, suggested the involvement of bacterial biofilms in chronic infection. We hypothesized and later demonstrated that biofilm formation on GSs in mice and humans occurred and enhanced colonization and typhoid carriage (Crawford et al., 2010).

The extracellular matrix (ECM) of these biofilms is composed of proteins, DNA, and polysaccharides and are primarily responsible for its recalcitrance to antimicrobial substances. Amyloid proteins are associated with Alzheimer’s disease in humans (Schnabel, 2010) and are also a component of the ECM of biofilms produced by numerous bacterial species (Pritchard et al., 2017). Curli fibers, amyloid proteins expressed by enteric bacteria including *E. coli* and *Salmonella enterica*, are a pivotal structural component of the biofilm ECM (Römling et al., 2000; Kikuchi et al., 2005; Hufnagel et al., 2013; Adcox et al., 2016). Curli genes are arranged in two divergent curli-specific gene (*csg*) operons with independent promoters: one contains the structural components CsgA and CsgB (*csgBAC*), and the second contains the regulator CsgD and other structural and facilitator proteins (*csgDEFG*). Curli fibers are detected by the immune system during urinary tract infections, sepsis, and gastrointestinal inflammation caused by *E. coli* or *S.* Typhimurium (Bian et al., 2000; Humphries et al., 2003; Kai-Larsen et al., 2010), leading to the expression of important cytokines and chemokines (Rapsinski et al., 2013; Gallo et al., 2015). The regulation of ECM production in *Salmonella* is complex, in part involving temperature and several transcription factors that culminate in CsgD expression: the direct regulator of curli genes as well as of other ECM components (e.g. cellulose). The colony Rdar (red, dry, and rough) phenotype is linked to curli and cellulose expression, and while most *S.* Typhimurium isolates are Rdar+, *S.* Typhi strains have been described to be Rdar- (smooth and white; Saw) because of a truncation the last 8 amino acids in the C-terminus of *S.* Typhi CsgD caused by a mutation that introduces a premature stop codon (Parkhill et al., 2001; MacKenzie et al., 2019). This mutation has also been ascribed to *S*. Typhi’s deminished biofilm forming ability (MacKenzie et al., 2019). A recent study has shown that *S*. Typhi CsgD loosely binds the *csgBAC* promotor and the introduction of an *S*. Typhimurium *csgD* gene does not rescue the Rdar colony morphotype in *S*. Typhi, additionally, they could not detect *S*. Typhi CsgD protein by Western Blot in the complemented strain (Ou et al., 2023). However, *S.* Typhi forms biofilms *in vitro* (Marshall et al., 2014; González et al., 2018; Devaraj et al., 2021; Hahn et al., 2021), and *in vivo* on human gallstones (Crawford et al., 2010). We hypothesize that biofilm regulation in *S*. Typhi is fundamentally different from that of its non-typhoidal counterparts. Therefore, we set out to investigate what role, if any, CsgD plays in biofilm formation in *S*. Typhi.

## Materials and methods

### Bacterial strains and growth conditions

Strains used in this study are listed in **Supplementary Table 1** in the **Supplementary Material**. Strains were streaked on Luria-Bertani (LB) agar plates and incubated at 37°C overnight. Single colonies were used to start overnight (O/N) liquid cultures. Planktonic cells were grown at 37°C on a rotating drum in tryptic soy broth (TSB). Strains containing the complementation plasmids pBR322, pWSK29 or both pRB322 and the luciferase reporter plasmid (pCS26) were supplemented with ampicillin (Amp; 100 μgml^-1^) and tetracycline (Tet; 15 μgml^-1^), ampicillin (Amp; 100 μgml^-1^) or kanamycin (Kan; 45 μgml-^1^), ampicillin (Amp; 100 μgml^-1^) and tetracycline (Tet; 15 μgml^-1^), respectively, to keep selective pressure on the plasmids.

### Generation of mutants and cloning procedures

Chromosomal mutations were constructed by the lambda Red mutagenesis method (Datsenko and Wanner, 2000). Oligonucleotide primers used to perform gene deletions, cloning, and site-directed mutagenesis are listed in **Supplementary Table 2**.

### Construction of complementation plasmids

Complementation plasmids were constructed using Gibson assembly (Gibson et al., 2009). Primers are listed in **Supplementary Table 2**.

### Congo Red and calcofluor white plate assays

Rdar morphotypes were assessed by normalizing O/N cultures grown in TSB to OD_600_ = 0.8 and washing cells twice with sterile ddH_2_O, followed by spotting 3 μL onto congo red plates (YESCA agar: 1 g/L yeast extract [Fisher BioReagents], 10 g/L Casamino Acids [Difco Laboratories], 15g/L agar [Fisher BioReagents]) supplemented with 40 μg/mL Congo red [Fisher Chemical] and 20 μg/mL Coomassie brilliant blue [Fisher BioReagents]). Cellulose production was assessed on LBNS containing 20 gml^-1^ calcofluor white (Fluorescent Brightener 28, Sigma-Aldrich, St. Louis, MO). Plates were incubated at either room temperature or 37°C for 4 days and then imaged. Calcofluor white plates were visualized under UV light at 366 nm.

### Western blot analysis

CsgA was detected in whole-cell lysates using a polyclonal antisera generated against *S*. Typhimurium CsgA (gift of Çagla Tükel, Temple University). Western blotting was conducted as previously described (Zhou et al., 2013). In brief, biofilms were grown for 4 days on congo red plates, as outlined above. Cells were scraped from agar plates, resuspended in 1 ml PBS (pH 7.4), and normalized to the lowest OD_600_ value. 150μL of normalized cells was pelleted, the supernatant was removed, and the pellet was resuspended in 70μL of 88% formic acid (Sigma-Aldrich, St. Louis, MO). Samples were dried using a DNA 110 SpeedVac Concentrator (Savant) and resuspended in 100μL Laemmli loading buffer. 15μL of each sample was loaded onto a 4-15% SDS-PAGE gel along with 10μL Bio-Rad Precision Plus Protein™ Unstained Protein Standard. Proteins were electrophoresed for 5 minutes at 50V, then the voltage was increased to 175V until the dye front reached the bottom of the gel. Proteins in the gel were transferred for 30 min at 1A and 25V to a methanol (MeOH)-activated polyvinylidene difluoride (PVDF) membrane and blocked with agitation for 1 h in 3% bovine serum albumin (BSA) in Tris-buffered saline with 0.1% Tween^®^ 20 detergent (TBST) at room temperature. The membranes were incubated with anti-CsgA antibody (1:10,000 in 3% BSA-TBST) overnight at 4°C, washed in TBST (5 washes, 5 min each), and incubated with horseradish peroxidase (HRP) conjugated goat anti-rabbit antibody (1:7,000 in 3% BSA-TBST for 1 h) at room temperature. The membranes were then washed in TBST (5 washes, 5 min each), incubated with Clarity™ Western ECL Substrate (Bio-Rad), and visualized using the Bio-Rad (Hercules, CA) ChemiDoc system.

### Luciferase reporter strain assays

For bioluminescence assays, reporter plasmids were introduced into the strains containing the complementation pBR322-based plasmid (**Supplementary Table 1**). The bioluminescence reporter plasmids (kindly provided by Aaron White) are based on pCS26 and have the promoter regions of either the *csgBAC* or *csgDEFG* curli operon cloned upstream of the *lux* genes (White et al., 2006). Overnight cultures grown in TSB were normalized to OD_600_ = 0.8 and diluted 1:100 in LBNS to a final volume of 200 μL in 96-well clear-bottom black plates (9520, Costar; Corning, Tewksbury, MA) covered with a Breathe-Easy sealing membrane (Sigma-Aldrich, St. Louis, MO). Cultures were assayed for luminescence (0.1 s) every 30 min for the first 16 hr, then every 24 hr at 25°C in a Spectramax M3 (Molecular Devices, Sunnyvale, CA) with shaking before every read. Each strain was assessed in 4 replicate wells and each experiment was repeated at least 3 times.

### Biofilm growth

*S*. Typhi biofilms were grown as follows. Cultures were grown in TSB at 37°C O/N on a rotating drum. The following day, cultures were normalized to OD_600_ = 0.8 and diluted 1:100 in TSB to a final volume of 200 μL in non-treated polystyrene 96-well plates (Corning). Plates were previously coated with 5 mg/mL cholesterol (Sigma) that had been dissolved in equal parts ethanol and isopropanol (Sigma) and evaporated in a biosafety cabinet. The plates were incubated for 4 days at 25°C on an LSE™ nutating mixer (Corning, Inc.) at 24 rpm. Growth medium was replenished every 48 h.

Attached biofilms of *S*. Typhi were washed twice in double-distilled water (ddH_2_O), heat-fixed for 1 h at 60°C, and stained with 0.33% crystal violet for 5 min. After two subsequent washes in ddH_2_O, the dye was eluted using 33% acetic acid, and the OD_570_ was measured in a SpectraMax spectrophotometer with SoftMax Pro software (Molecular Devices) to determine the amount of dye retained, which correlates to the amount of biofilm present. All biofilm experiments were performed in triplicate.

### Statistical analysis

All experiments were performed with three or more biological replicates and repeated at least three times. Only groups with comparable treatments were statistically analyzed. Specific statistical analysis is described in the figure legends and was performed using the software GraphPad Prism version 10.2.3. Statistical significance was represented as follows: ns, not significant; *, p ≤ 0.05; **, p ≤ 0.01; ***, p ≤ 0.001; and ****, p ≤ 0.0001.

## Results

### Complementation of functional CsgD in *S*. Typhi under its native promoter

**Figure 1:**
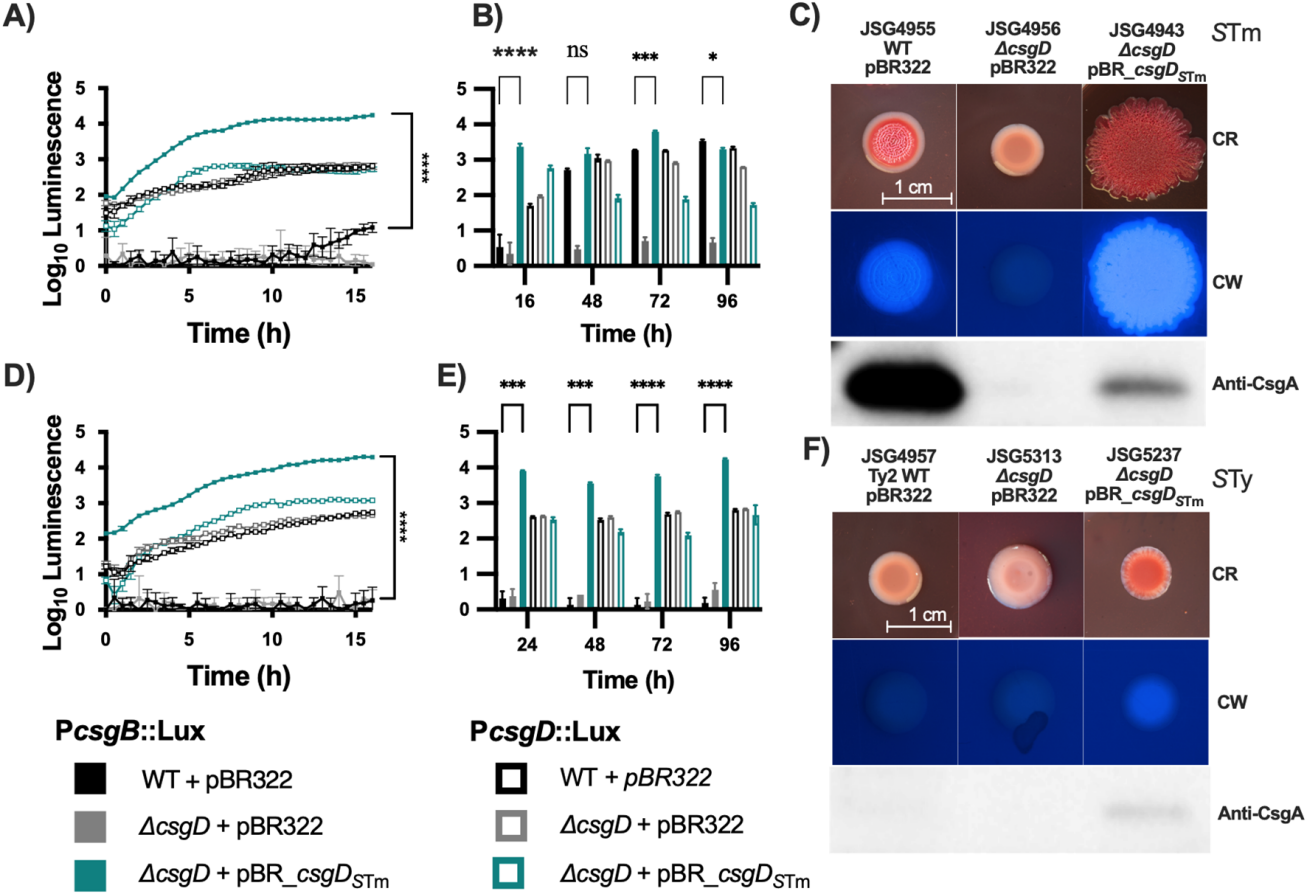
Complementation of *csgD* in laboratory strains. The *S*. Typhimurium 14028 *csgD* gene was cloned into pBR322 and used to complement *S*. Typhi; *S*. Typhimurium 14028 was used as the control. To verify that the complementation plasmid was inducing transcription of target genes, it was cloned into strains containing the pCS26 reporter plasmids that contain the *csgDEFG* or *csgBAC*, promoter sequences from S.Typhimurium 14028 fused to *luxCDABE*. **(A, D)** are kinetic analysis of the first 16 hrs of activity in *S*. Typhimurium and S. Typhi, respectively. **(B, E)** are daily measurements. **(C, F)** Strains were spotted on congo red (CR) or calcofluor white (CW) plates to check for Rdar and cellulose production, respectively. Cells from CR plates were scraped and used for Western Blot to detect CsgA. Bar charts are presented as mean with standard deviation (SD). Representative charts are shown from one experiment performed with at least four biological replicates and repeated at least three times with similar results. Total luminescence counts were log transformed and compared using two-way ANOVA followed by Tukey’s multiple-comparison test (ns, not significant; *, P ≤ 0.05; ***, P ≤ 0.001; ****, P ≤ 0.0001).

Since it has been proposed that *S.* Typhi has a reduced capacity to aggregate and produce biofilms due to a point mutation that causes a truncation of the CsgD regulator (MacKenzie et al., 2019) we introduced a copy of the *S*. Typhimurium *csgD* (*csgD_S_*_Tm_) gene into *S.* Typhi by cloning the gene under its own promoter in the low-copy number plasmid pBR322. As a control, we also tested the efficacy of complementation by introducing the plasmid into a *S*. Typhimurium *ΔcsgD* strain. We first checked the effect of the plasmid contruct on *csgBAC and csgDEFG* expression in *S*. Typhimurium and *S*. Typhi *csgD* mutants, by measuring the activity of *lux* reporter plasmids (White et al., 2006). In line with previous findings, the *csgDEF* promoter is not regulated by CsgD (Römling et al., 2000) and maintains a steady expression regardless of *csgD* mutation or corrective complementation in both *S*. Typhimurium and *S.* Typhi in the first 16 hrs of growth (**Figures 1A, D**). Remarkably, *csgDEF* expression seems to decline in later time points in *S*. Typhimurium but not *S*. Typhi (**Figures 1B, E**). Conversely, the introduction of a plasmid-borne *csgD_S_*_Tm_ increased *csgBAC* expression by 3 logs in *ΔcsgD S*. Typhimurium at 16hrs and was maintained at levels comparable to the wild type (WT) *S*. Typhimurium in later measurements (**Figures 1A, B**). In *S*. Typhi, *csgBAC* is not expressed in the WT strain at any time point, but complementation with plasmid-borne *csgD_S_*_Tm_ increased the expression of this operon dramatically (**Figures 1D, E**).

Curiously, at a phenotypic level, plasmid complementation of CsgD in *S*. Typhimurium caused an exaggerated Rdar colony morphotype with increased cellulose production but a decrease in CsgA protein levels (**Figure 1C**). WT *S*. Typhi strains have a Saw colony morphology and are cellulose negative, as observed on calcofluor white plates. Complementation of *csgD* in the laboratory strain Ty2 (JSG4383) with the plasmid-borne *csgD_S_*_Tm_ resulted in colonies that are red (indicating Congo Red binding) and cellulose-positive, and in which CsgA can be weakly detected (**Figure 1F**). In conclusion, complementation of *S*. Typhi with *csgD_S_*_Tm_ partly restores cellulose and curli production but does not restore the characteristic Rdar colony morphotype of *S*. Typhimurium.

**Figure 2:**
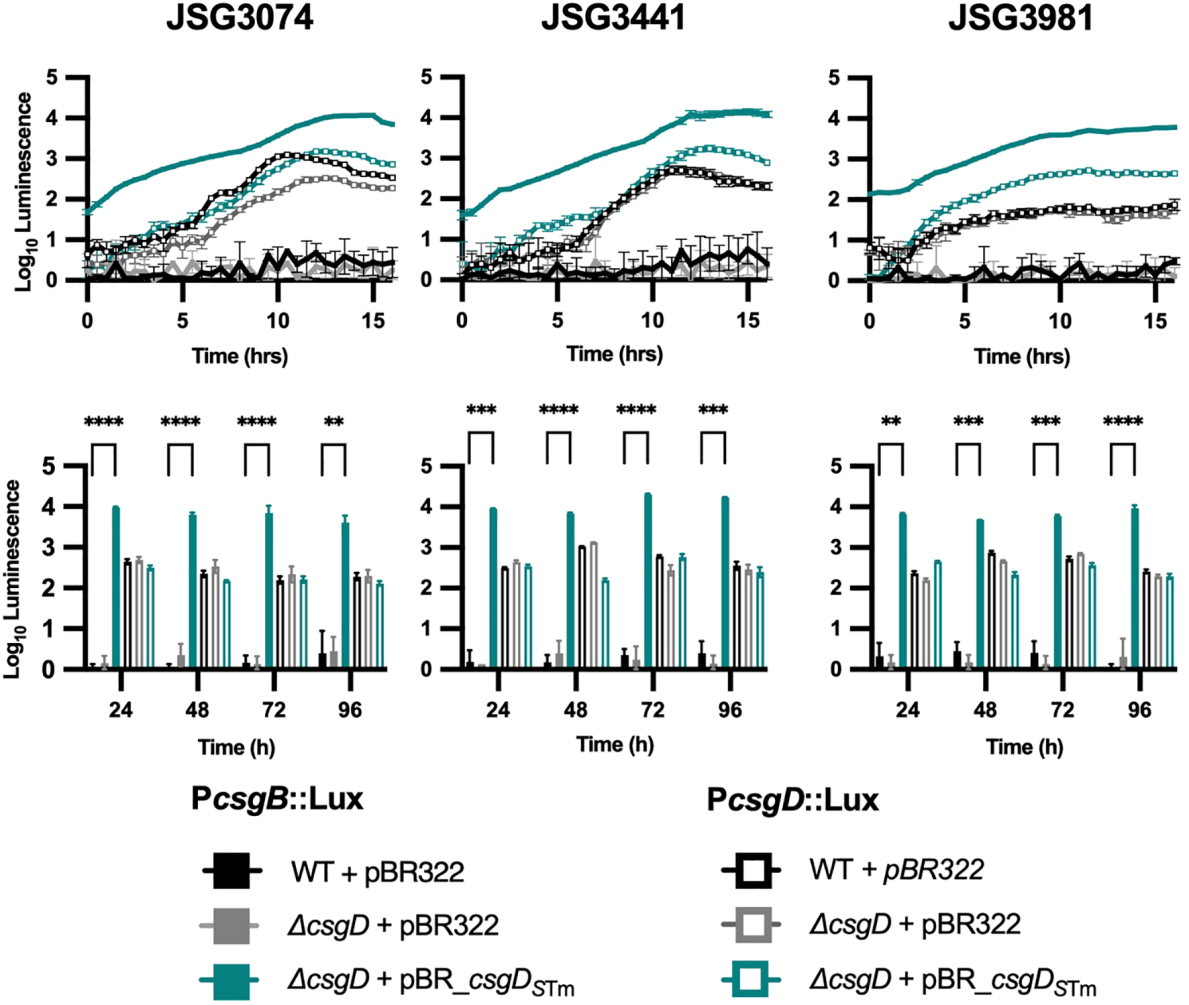
Curli operon expression in clinical isolates. Activity of *csgBAC* or *csgDEFG* promoters (P) cloned into the pCS26::*luxCDABE* reporter plasmid in representative clinical isolate background strains. The top row shows 16 hr kinetic assays and the bottom are daily measurements. A two-way ANOVA followed by Tukey’s multiple-comparison test were used to compare the different conditions (**, P ≤ 0.01; ***, P ≤ 0.001; ****, P ≤ 0.0001).

We know from experience that *S.* Typhi has the capacity to form biofilms *in vitro* (Marshall et al., 2014; González et al., 2018; Hahn et al., 2021), and *in vivo* on human gallstones (Crawford et al., 2010). We therefore decided to test whether complementation with *csgD_S_*_Tm_ could induce expression of curli genes in *S.* Typhi clinical isolates. The strains chosen were isolated from chronic GB carriers, which we know are proficient biofilm formers, as well as isolates from acute Typhoid cases (**Supplementary Table 1**) (Devaraj et al., 2021). Overall, the selected strains behaved similarly to the laboratory strain *S*. Typhi Ty2 (JSG4383; **Figure 1D**). With regard to *csg* gene expression, profiles of the *csgBAC* (induced by *csgD_S_*_Tm_ expression) and *csgDEF* (not induced by *csgD_S_*_Tm_ expression) operons were almost identical among the clinical strains examined, with the only noticeable difference being a slightly lower *csgDEF* expression in JSG3981 at early time points (**Figure 2**).

**Figure 3:**
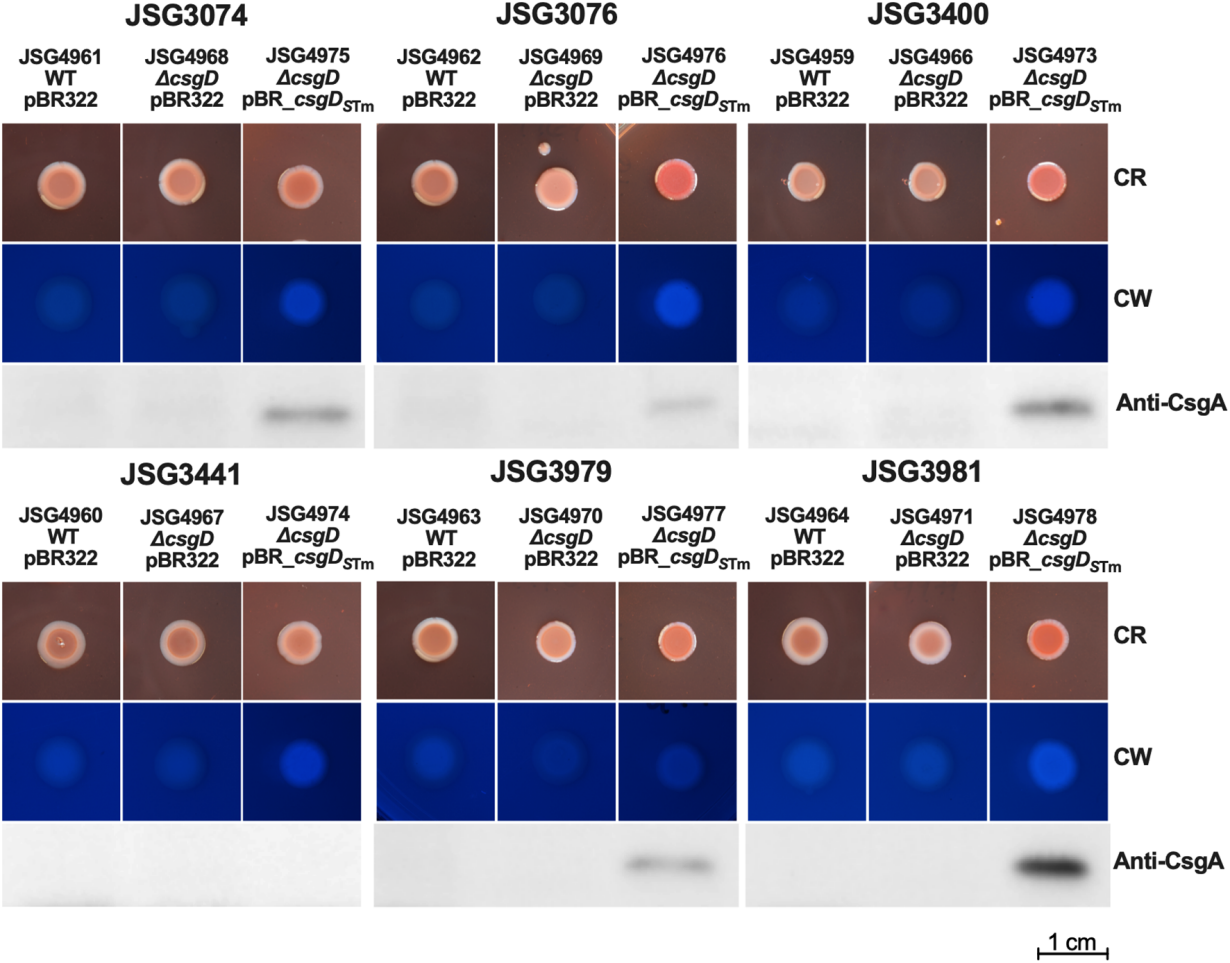
Clinical Strains were transformed with empty vector (control) or the *csgD_STm_*-complementing plasmid and spotted on congo red (CR) or calcofluor white (CW) plates to check for Rdar and cellulose production, respectively. Cells from CR plates were scraped and used for Western Blot to detect CsgA.

Phenotypically, the story was also similar; clinical strains complemented with *csgD_S_*_Tm_ showed red colonies, were cellulose-positive, and CsgA could be detected in all but strain JSG3441 (**Figure 3**). As expected, the empty vector, Δ*csgD*, and WT strain controls were phenotypically Saw and did not express CsgA. In conclusion, the clinical *S*. Typhi strains, which produce more robust biofilms than the laboratory strain, behaved similarly after complementation with a functional *csgD* gene, indicating a different role for the biofilm regulator in *S*. Typhi versus non-typhoidal strains.

### Complementation of CsgD under a constitutive promoter

**Figure 4:**
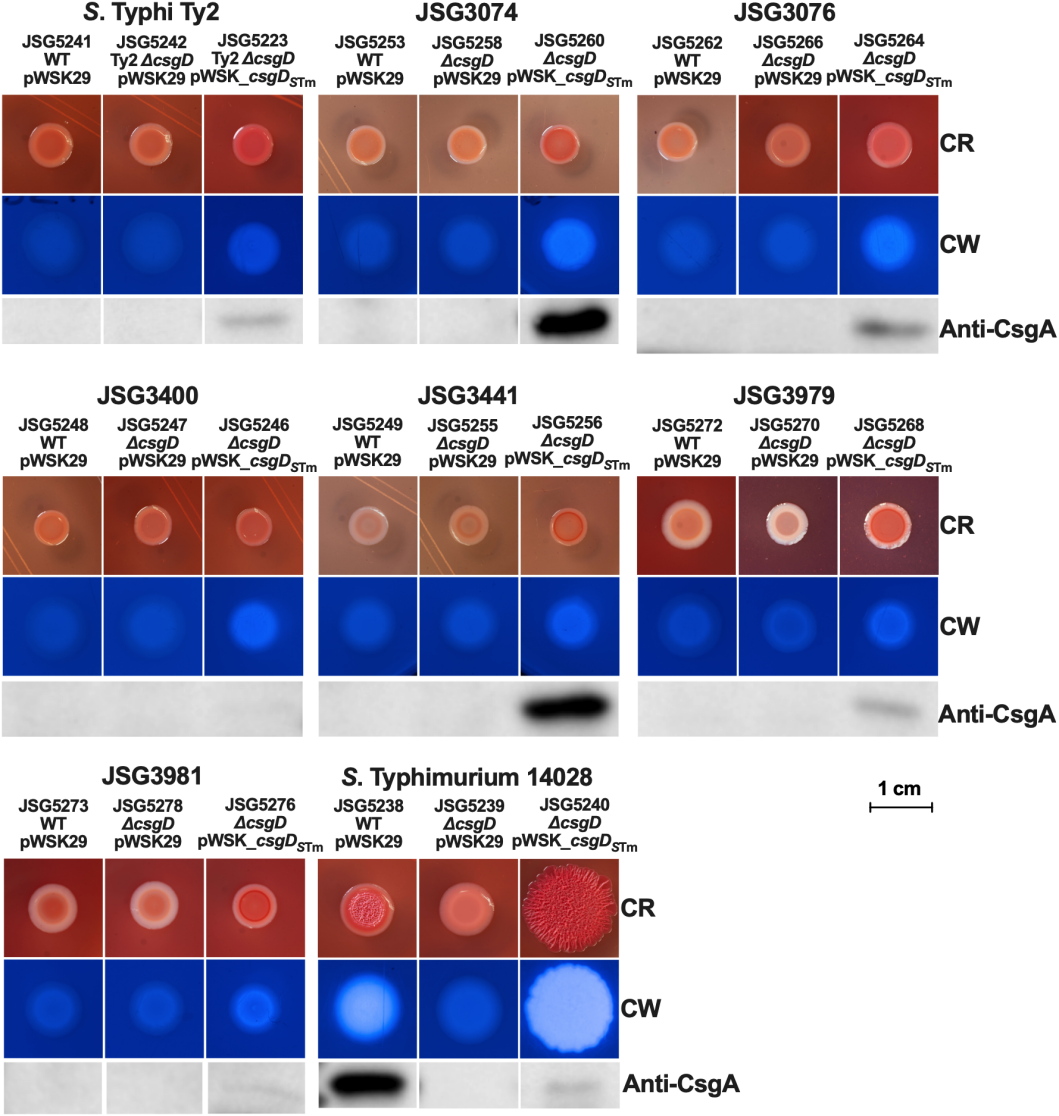
Strains (WT and Δ*csgD* mutants) were transformed with the empty vector (control) or the *csgD_STm_*-complemented plasmid and spotted on congo red (CR) or calcofluor white (CW) plates to check for Rdar and cellulose production, respectively. Cells from CR plates were scraped and used for Western Blot to detect CsgA.

A potential hypothesis for why *S*. Typhi doesn’t make Rdar colonies could be that the *csgDEFG* operon promoter could be repressed by a novel *S*. Typhi regulatory protein. To address this possibility, we created a pWSK_*csgD_S_*_Tm_ plasmid where *csgD_S_*_Tm_ is under the constitutive *lac* promoter. Upon analysis, the results were very similar to those observed with the pBR*_csgD_S_*_Tm_ plasmid and the native promoter, as the complemented *S*. Typhimurium strain presented an exaggerated Rdar colony, increased cellulose production, and reduced CsgA production, while the Δ*csgD* strain was deficient in cellulose and *csgA*/curli production as expected. The S. Typhi *ΔcsgD* complemented strains showed a noteworthy difference with this plasmid. We observed levels of CsgA production in strains JSG3074 and JSG3441 comparable to the *S*. Typhimurium wild type. Otherwise, they exhibited the same colonies with an increase in red color, a rescue of modest levels of cellulose, but no Rdar colonies in any strain (**Figure 4**), similar to what was observed with the *csgD_S_*_Tm_ plasmid with the native promoter (**Figure 3**). We also complemented our laboratory strains and a select group of our clinical isolates with a copy of *S.* Typhi *csgD* (*csgD_S_*_Ty_) constitutively expressed in the pWSK29 plasmid. This construct was not able to complement Rdar, cellulose, or CsgA production in any of the strains tested (**Supplementary Figure 1**). We also introduced pWSK_*csgD_S_*_Tm_ into a Vi antigen mutant, with the rationale that the Vi antigen could interfere with curli expression; however, we did not see any differences in comparison to the clinical isolate complemented strains mediated by the pWSK_*csgD_S_*_Tm_ plasmid in the Vi antigen deficient genetic background (Data not shown). In conclusion, the lack of Rdar complementation in *S*. Typhi does not seem to be associated to repression by an upstream transcriptional regulator or interference by the Vi antigen.

### Effects of CsgD complementation on biofilm formation in an *in vitro* GB/GS model

**Figure 5:**
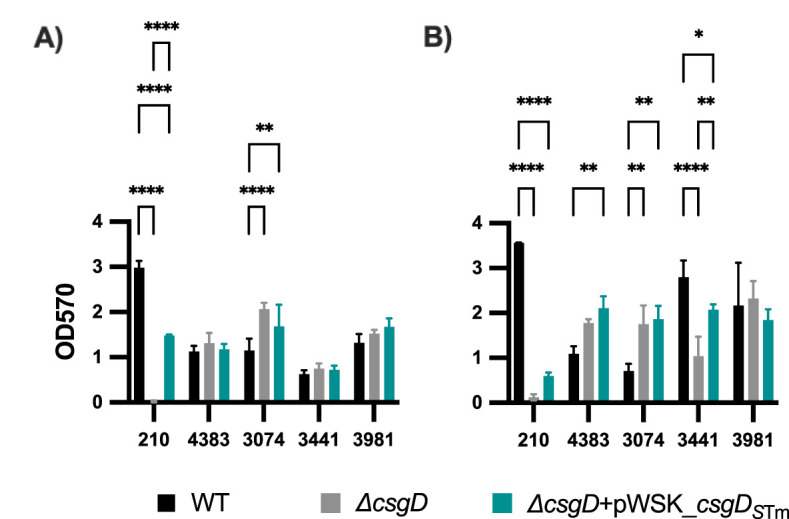
Biofilm formation of complemented *S*. Typhimurium and *S*. Typhi *ΔcsgD* strains. Bacteria were grown in cholesterol-coated 96-well plates in 200 μL of **(A)** tryptic soy broth (TSB) or **(B)** TSB with 1% ox bile. Biofilms were stained with crystal violet for relative biofilm measurement as determined at an optical density at 570 nm (OD_570_). Bar charts presented as mean with standard deviation (SD). Statistical analyses were performed using a two-way analysis of variance (ANOVA) with Dunnett’s multiple comparison test. *, P, 0.05; **, P, 0.01; ****, P, 0.0001. Representative charts are shown from one experiment performed with at least four biological replicates and repeated at least three times with similar results.

CsgD is a major biofilm regulator in *E. coli* and *Salmonella*, and curli has been shown to be perhaps the most important biofilm matrix component (Römling et al., 2000; Kikuchi et al., 2005; Hufnagel et al., 2013; Adcox et al., 2016). While the Rdar colony morphology has been used extensively in the field to analyze curli production and biofilm-forming ability, it does not reflect the entire essence of a biofilm. In line with this, we tested biofilm formation on 96 well plates using the WT, *Δcsg*, and *ΔcsgD* + pWSK_*csgD_S_*_Tm_ strains in various representative backgrounds. Consistent with what we previously reported (Adcox et al., 2016), CsgD is absolutely critical for biofilm formation in *S*. Typhimurium, as biofilm levels in our *S*. Typhimurium *ΔcsgD* strain are nearly background levels on cholesterol-coated plates with (**Figure 5A**) or without (**Figure 5B**) bile. Complementation of the *ΔcsgD* strain with pWSK_*csgD_S_*_Tm_ rescues the biofilm phenotype in *S*. Typhimurium, but not completely (**Figures 5A, B**). This is consistent with the complemented strain’s ability to regain the Rdar morphotype, cellulose and CsgA expression. In *S.* Typhi, the biofilm results are much more nuanced. Several *S*. Typhi strains are very capable biofilm producers, especially in GB conditions (cholesterol+bile; **Figure 5B**). Though somewhat variable strain-to-strain, the *ΔcsgD* and *ΔcsgD* complemented strains are not generally statistically significantly different in biofilm-forming ability compared to the parental WT. In conclusion, deletion, or complementation of the transcriptional regulator CsgD in *S*. Typhi does not dramatically affect biofilm forming ability, especially when compared to its effect in *S*. Typhimurium.

## Discussion

Investigators have proposed that *S*. Typhi has a decreased ability to form biofilms due to one or more of the following traits: (1) host specialization, as specialized pathogens tend to lose traits that are not vital in the host environment (Merhej et al., 2009; Murray et al., 2020); (2) form of transmission, as *S*. Typhi is not readily found in any animal vector or the environment, where biofilms provide distinct survival advantages (White et al., 2006; Steenackers et al., 2012), but rather relies on human-to-human transmission; and (3) the stealth strategy that it uses once inside the host, as it has been proposed that since biofilm components tend to activate host immune defenses (Bian et al., 2000; Humphries et al., 2003; Kai-Larsen et al., 2010; Rapsinski et al., 2013; Gallo et al., 2015).

To discover whether the differences in biofilm formation observed in *S*. Typhi versus non-typhoidal strains were due to a simple point mutation that renders the biofilm regulator CsgD nonfunctional, we introduced a copy of the *S*. Typhimurium *csgD* gene into several *S*. Typhi strains on low copy plasmids. We then tested whether this corrective substitution could make *S*. Typhi recapitulate the biofilm characteristics of *S*. Typhimurium, namely Rdar colony morphology, curli fimbriae production, and cellulose production. Additionally, we tested biofilm proficiency in our *in vitro* GB model. CsgD is part of the RpoS regulon (Olsén et al., 1993; Robbe-Saule and Norel, 1999) and is activated during stationary phase (after about 5-6 hours of growth at 37°C) in *S.* Typhimurium grown in rich media. A few hours into stationary phase, there is an increase in *csgDEFG* promoter activity in both typhoidal and non-typhoidal serovars (**Figures 1A, D**), which is expected as this promoter is activated independently of CsgD (Römling et al., 2000). The activity was similar across different genetic backgrounds (**Figures 1A, D**).

The introduction of *csgDS*Tm into *S.* Typhi significantly induced *csgBAC* operon expression compared to the WT and Δ*csgD* strains. Interestingly, expression in both serovars containing the *csgDS*Tm complementation plasmid began early, unlike the WT *S.* Typhimurium, where activity was only observed in stationary phase (**Figures 1A, D**). This suggests that *S.* Typhi CsgD is unable to induce *csgBAC* expression, likely due to its truncation and instability (Ou et al., 2023).

The *csgD_STm_* complemented *S*. Typhimurium *ΔcsgD* strain exhibited interesting behavior on congo red and calcofluor white plates. The complemented *S*. Typhimurium *ΔcsgD* grew a much bigger colony than the WT strain in the same amount of time, and the colony was also more rugose and brighter when observed under UV light on calcofluor white plates, indicating higher cellulose production. Unexpectedly, when this colony was scraped from the plate for Western Blot analysis, there was much less CsgA protein detected than in the WT strain. The larger size of the colony could be due to the increase in cellulose synthesis. The apparent decrease in CsgA protein was a surprising result, especially since expression of the *csgBAC* operon increased considerably (**Figure 1A**). There could be various reasons for the observed CsgA reduction. The intergenic region between the two *csg* operons is one of the largest in the *Salmonella* genome (Barnhart and Chapman, 2006) and also one of the most regulated with OmpR, MlrA, IHF, RstA, CpxR, H-NS all shown to bind and regulate its activation (Ogasawara et al., 2010). The different curli components perform various functions and exhibit complex interactions. For example, the nucleator protein CsgB anchors the CsgA polymer to the cell, but also forms biomolecular condensates with CsgF, which are necessary for CsgA polymerization. Additionally, CsgF regulates CsgA secretion (Swasthi et al., 2023). The increased *csgD* expression and early expression of the *csgBAC* operon (**Figure 1A**) may disrupt the finely tuned regulation of these components. Curli fiber assembly could also be affected if monomers are secreted early without being assembled and disseminate into the surrounding agar.

Our rationale for making the plasmid construct was to test if a non-mutated *csgD* could induce *S*. Typhi curli and cellulose production and complement the lack of the Rdar morphotype. While both curli and cellulose production were partially restored, it was ultimately not enough expression to induce a Rdar morphotype. Perhaps the levels were not enough to reach the threshold necessary for a Rdar colony. Our clinical isolates behaved similarly (**Figures 2**, **3**), even though they possessed variable biofilm-forming capabilities (some isolated from chronic carriers that formed robust biofilms) (Devaraj et al., 2021). Interestingly, CsgA could not be detected in one of our acute case isolates (JSG3441) but was detected all other *S*. Typhi strains (**Figure 3**; **Supplementary Table 1**).

In parallel, we designed a plasmid construct with *csgD_S_*_Tm_ under the constitutive *lac* promoter to circumvent any potential *csgD* repressors in *S*. Typhi. This plasmid construct behaved very similarly to the *csgD_S_*_Tm_ construct with its own promoter (in the pBR322 plasmid) and didn’t induce a Rdar colony morphology in any of the tested *S*. Typhi strains (**Figure 4**). Interestingly, we observed a high production of CsgA in two strains: JSG3074 (a gallstone isolate) and JSG3441 (a stool isolate). However, JSG3441 showed no CsgA production with the pBR322 plasmid complementation, highlighting the complex biofilm behavior of *S*. Typhi. We didn’t measure promoter expression as pWSK29 is not compatible with the luciferase reporter plasmid, but after observing the similarities in induced phenotypes between the two *csgD_S_*_Tm_ constructs, we didn’t consider it necessary.

A recent study (Ou et al., 2023) also complemented *S*. Typhi with *S*. Typhimurium *csgD* and also deleted and complemented the entire *ΔcsgBACDEFG S*. Typhi genetic cluster with the *S*. Typhimurium locus. Neither of these strategies induced an Rdar phenotype, ruling out the possibility of the other components of the curli machinery being non-functional in *S*. Typhi. Additionally, they could not detect the *S.* Typhi CsgD protein by Western Blot except when *S.* Typhi was complemented with the *csgD_S_*_Tm_ construct, indicating that the protein might be degraded in *S*. Typhi. These results would rule out concerns of overexpression of *csgD* when using our pBR322 and pWSK29 plasmids, as they had similar results with a single copy expression.

Our research focuses heavily on chronic typhoid carriage, which is primarily associated with the GB and is highly correlated with biofilm formation on cholesterol GSs. Consequently, we have historically focused on *in vitro* biofilm systems that model this environment with cholesterol-coated surfaces and bile. The Rdar morphotype has been used extensively as a model for *E. coli* and *Salmonella* biofilm research (Römling et al., 1998; Gibson et al., 2006), and while useful, it may not accurately reflect the GS surface. For this reason, we sought to determine how the complementation of *S*. Typhi *csgD* would translate to our *in vitro* models. Interestingly complementation with pWSK_*csgD_S_*_Tm_ (*lac* promoter) doesn’t fully complement biofilm formation of a *S*. Typhimrium *ΔcsgD* mutant strain to WT levels. This may be explained in part by the result from our Western Blot that showed decreased CsgA levels in this strain (**Figure 4**). In *S*. Typhi, CsgD does not seem to play a crucial role in biofilm formation (**Figure 5**). As we have previously observed (Devaraj et al., 2021), there is a great variability in the biofilm-forming capacity of *S*. Typhi strains, but these attributes appear to be independent of CsgD. Moreover, significant variation in RpoS levels between *S*. Typhi isolates has also been observed (MacKenzie et al., 2019) indicating uniquely heterogenous biofilm behavior between strains. Moreover, Rpos has recently been found to be essential for the transcription of S. Typhi biofilm genes both in vitro and in vivo, as demonstrated in a zebrafish model of chronic gallbladder carriage (Desai et al., 2023).

CsgD acts as a bistable switch between planktonic and biofilm in *S*. Typhimurium, which has been proposed as a hedge-betting strategy that shifts the population to either phenotype as a safeguard against rapid changes in the environment (Grantcharova et al., 2010; MacKenzie et al., 2015). Our data would suggest that CsgD doesn’t play an important role in *S*. Typhi biofilm formation versus other *Salmonella* serovars and in *E. coli*. However, it is interesting that *S*. Typhi, known for the plasticity of its genome, which has gone through significant rearrangements and gene losses (Liu and Sanderson, 1995, 1996; McClelland et al., 2004; Sabbagh et al., 2010), has kept the curli genes basically intact (beside the point mutation in *csgD*). In fact, the highly regulated promoter region between the operons is identical to *S*. Typhimurium. Additionally, antigens to curli components have been identified in *S*. Typhi patients, suggesting curli production during infection, interestingly these included CsgE, CsgF, and CsgG, but not CsgA, CsgB, or CsgD (Harris et al., 2006). This bolsters the hypothesis that *S*. Typhi prioritizes immune evasion over hedge-betting strategies.

While *S*. Typhi can form biofilms, it doesn’t do so as prolifically as *S*. Typhimurium *in vitro*. It is possible that *S*. Typhi has devised alternative mechanisms that allows it to form biofilms in the very niche gallbladder environment but remain undetected in the host. This presents the question: if the mechanism by which *S*. Typhi dampens curli and other ECM components is through decreased CsgD activity due to a point mutation, why does the re-introduction of a fully functional *csgD* gene not fully restore biofilm properties (*i.e*., Rdar)? Post-transcriptional or translational regulation could be a possibility, especially since the promoter regions in *S*Tm and *S.* Typhi are identical. Alternatively, the system could be shut off by an unidentified repressor or the truncated CsgD could be unstable and/or targeted for degradation (Ou et al., 2023). These questions will be the subject of future research.

In summary, the *csgD* gene in *S*. Typhi has a point mutation that introduces an early stop codon that results in the truncation of the last 8 amino acids in the C-terminus. Complementation with a fully functional *csgD* gene from *S*. Typhimurium partially restored production of cellulose and CsgA protein, although it was not enough to restore the Rdar morphotype, a hallmark of aggregative behavior in *S*. Typhmiurium and *E*. *coli*. Additionally, CsgD does not seem to play a crucial role in biofilm formation in *S*. Typhi compared to other members of *Salmonella*, as *S*. Typhi wild type, *csgD* mutant, and *csgD* overexpressing strains had similar biofilm-forming capacity. These results suggest that *S.* Typhi has evolved a biofilm formation strategy distinct from related serovars. Understanding the mechanisms behind this process could lead to new therapeutic approaches for treating chronic carriers.

## Data Availability

Requests to access the datasets should be directed to Juan.GonzalezParedes@nationwidechildrens.org.
