# Supplementary figures and images for: Functional role of the biofilm regulator CsgD in Salmonella enterica sv. Typhi
Source: Front Cell Infect Microbiol. 2024 Dec 11;14:1478488. doi: 10.3389/fcimb.2024.1478488 (PMC11668344; doi:10.3389/fcimb.2024.1478488)

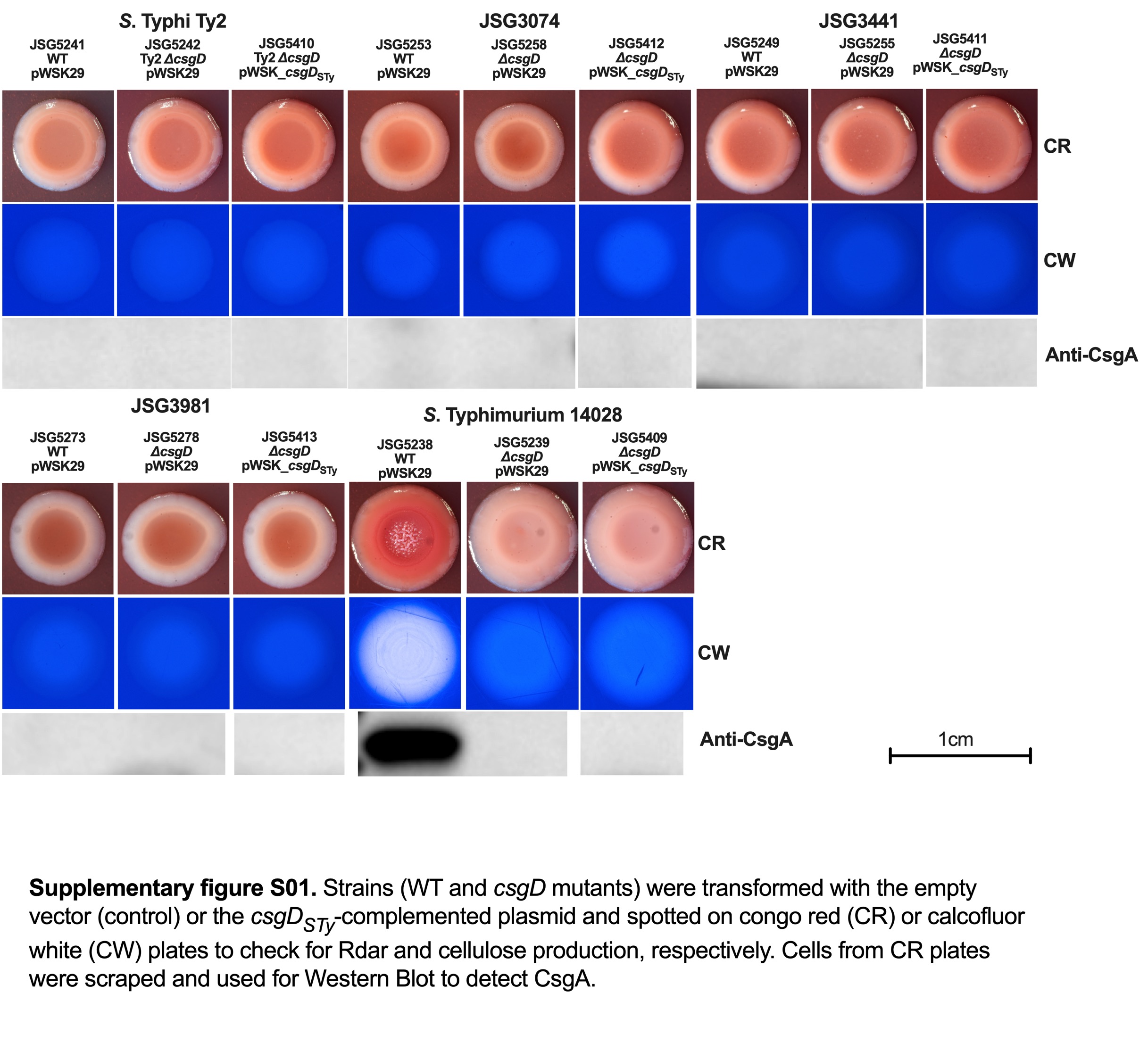

Supplement: Supplementary file 1 [file Image1.jpeg]
