# Supplementary material for: Functional role of the biofilm regulator CsgD in Salmonella enterica sv. Typhi
Source: Front Cell Infect Microbiol. 2024 Dec 11;14:1478488. doi: 10.3389/fcimb.2024.1478488 (PMC11668344; doi:10.3389/fcimb.2024.1478488)
Supplement: Supplementary file 2 [file Table1.docx]

**Supplementary Table 1.** Strains and plasmids used in this study.

| **Strains** | **Genotype or relevant phenotype** | **Reference or source** |
| --- | --- | --- |
| *S*. Typhimurium |  |  |
| JSG210 | Wild-type ATCC 14028 | ATCC |
| JSG4955 | WT JSG210 + pBR322 | This study |
| JSG5397 | WT JSG210 + pBR322 + pBA | This study |
| JSG5398 | WT JSG210 + pBR322 + pDEFG | This study |
| JSG5238 | WT JSG210 + pWSK29 | This study |
| JSG4227 | WT JSG210 Δ*csgD* | This study |
| JSG4956 | Δ*csgD*JSG4277 + pBR322 | This study |
| JSG5399 | Δ*csgD* JSG4277 + pBR322 + pBA | This study |
| JSG5400 | Δ*csgD* JSG4277 + pBR322 + pDEFG | This study |
| JSG4943 | Δ*csgD* JSG4277 + pBR_*csgD*_STm_ | This study |
| JSG5395 | Δ*csgD* JSG4277 + pBR_*csgD*_STm_ + pBA | This study |
| JSG5396 | Δ*csgD* JSG4277 + pBR_*csgD*_STm_ + pDEFG | This study |
| JSG5239 | Δ*csgD* JSG4277 + pWSK29 | This study |
| JSG5240 | Δ*csgD* JSG4277+ pWSK_*csgD*_STm_ | This study |
| JSG5409 | Δ*csgD* JSG4277+ pWSK_*csgD*_STy_ | This study |
|  |  |  |
| *S*. Typhi |  |  |
| JSG4383 | WT *rpoS+* | (Santander et al., 2007) |
| JSG4957 | WT JSG4383 + pBR322 | This study |
| JSG5403 | WT JSG4383 + pBR322 + pBA | This study |
| JSG5404 | WT JSG4383 + pBR322 + pDEFG | This study |
| JSG5241 | WT JSG4383 + pWSK29 | This study |
| JSG4399 | WT JSG4383 Δ*csgD* | This study |
| JSG5313 | Δ*csgD* JSG4399 + pBR322 | This study |
| JSG5405 | Δ*csgD* JSG4399 + pBR322 + pBA | This study |
| JSG5406 | Δ*csgD* JSG4399 + pBR322 + pDEFG | This study |
| JSG5237 | Δ*csgD* JSG4399 + pBR322_*csgD*_STm_ | This study |
| JSG5407 | Δ*csgD* JSG4399 + pBR322_*csgD*_STm_ + pBA | This study |
| JSG5408 | Δ*csgD* JSG4399 + pBR322_*csgD*_STm_ + pDEFG | This study |
| JSG5242 | Δ*csgD* JSG4399 + pWSK29 | This study |
| JSG5223 | Δ*csgD* JSG4399 + pWSK_*csgD*_STm_ | This study |
| JSG5410 | Δ*csgD* JSG4399 + pWSK_*csgD*_STy_ | This study |
| JSG3400 | Bile fluid clinical isolate | Ohio Department of Health |
| JSG4959 | JSG3400 + pBR322 | This study |
| JSG5248 | JSG3400 + pWSK29 | This study |
| JSG4885 | JSG3400 Δ*csgD* | This study |
| JSG4966 | JSG3400 Δ*csgD* JSG4885+ pBR322 | This study |
| JSG4973 | JSG3400 Δ*csgD* JSG4885+ pBR322_*csgD*_STm_ | This study |
| JSG5247 | JSG3400 Δ*csgD* JSG4885+ pWSK29 | This study |
| JSG5246 | JSG3400 Δ*csgD* JSG4885 + pWSK_*csgD*_STm_ | This study |
| JSG3441 | Stool clinical isolate | Ohio Department of Health |
| JSG4960 | JSG3441 + pBR322 | This study |
| JSG5450 | JSG3441 + pBR322 + pBA | This study |
| JSG5451 | JSG3441 + pBR322 + pDEFG | This study |
| JSG5249 | JSG3441 + pWSK29 | This study |
| JSG4886 | JSG3441 Δ*csgD* | This study |
| JSG4967 | JSG3441 Δ*csgD* JSG4886 + pBR322 | This study |
| JSG5452 | JSG3441 Δ*csgD* JSG4886 + pBR322 + pBA | This study |
| JSG5453 | JSG3441 Δ*csgD* JSG4886 + pBR322 + pDEFG | This study |
| JSG4974 | JSG3441 Δ*csgD* JSG4886 + pBR322_*csgD*_STm_ | This study |
| JSG5454 | JSG3441 Δ*csgD* JSG4886 + pBR322_*csgD*_STm_ + pBA | This study |
| JSG5455 | JSG3441 Δ*csgD* JSG4886 + pBR322_*csgD*_STm_ + pDEFG | This study |
| JSG5255 | JSG3441 Δ*csgD* JSG4886 + pWSK29 | This study |
| JSG5256 | JSG3441 Δ*csgD* JSG4886 + pWSK_*csgD*_STm_ | This study |
| JSG5411 | JSG3441 Δ*csgD* JSG4886 + pWSK_*csgD*_STy_ | This study |
| JSG3074 | Gallstone clinical isolate | General Hospital of Mexico, Mexico City |
| JSG4961 | JSG3074 + pBR322 | This study |
| JSG5432 | JSG3074 + pBR322 + pBA | This study |
| JSG5433 | JSG3074 + pBR322 + pDEFG | This study |
| JSG5253 | JSG3074 + pWSK29 | This study |
| JSG4887 | JSG3074 Δ*csgD* | This study |
| JSG4968 | JSG3074 Δ*csgD* JSG4887 + pBR322 | This study |
| JSG5434 | JSG3074 Δ*csgD* JSG4887 + pBR322 + pBA | This study |
| JSG5435 | JSG3074 Δ*csgD* JSG4887 + pBR322 + pDEFG | This study |
| JSG4975 | JSG3074 Δ*csgD* JSG4887 + pBR322_*csgD*_STm_ | This study |
| JSG5436 | JSG3074 Δ*csgD* JSG4887 + pBR322_*csgD*_STm_ + pBA | This study |
| JSG5437 | JSG3074 Δ*csgD* JSG4887 + pBR322_*csgD*_STm_ + pDEFG | This study |
| JSG5258 | JSG3074 Δ*csgD* JSG4887 + pWSK29 | This study |
| JSG5260 | JSG3074 Δ*csgD* JSG4887 + pWSK29_*csgD*_STm_ | This study |
| JSG5412 | JSG3074 Δ*csgD* JSG4887 + pWSK29_*csgD*_STy_ | This study |
| JSG3076 | Gallbladder tissue clinical isolate | General Hospital of Mexico, Mexico City |
| JSG4962 | JSG3076 + pBR322 | This study |
| JSG5262 | JSG3076 + pWSK29 | This study |
| JSG4888 | JSG3076 Δ*csgD* | This study |
| JSG4969 | JSG3076 Δ*csgD* JSG4888 + pBR322 | This study |
| JSG4976 | JSG3076 Δ*csgD* JSG4888 + pBR322_*csgD*_STm_ | This study |
| JSG5266 | JSG3076 Δ*csgD* JSG4888 + pWSK29 | This study |
| JSG5264 | JSG3076 Δ*csgD* JSG4888 + pWSK_*csgD*_STm_ | This study |
| JSG3979 | Gallbladder chronic carrier isolate | Gift of S. Baker (Vietnam) |
| JSG4963 | JSG3979 + pBR322 | This study |
| JSG5456 | JSG3979 + pBR322 + pBA | This study |
| JSG5457 | JSG3979 + pBR322 + pDEFG | This study |
| JSG5272 | JSG3979 + pWSK29 | This study |
| JSG4889 | JSG3979 Δ*csgD* | This study |
| JSG4970 | JSG3979 Δ*csgD* JSG4889 + pBR322 | This study |
| JSG5458 | JSG3979 Δ*csgD* JSG4889 + pBR322 + pBA | This study |
| JSG5459 | JSG3979 Δ*csgD* JSG4889 + pBR322 + pDEFG | This study |
| JSG4977 | JSG3979 Δ*csgD* JSG4889 + pBR322_*csgD*_STm_ | This study |
| JSG5460 | JSG3979 Δ*csgD* JSG4889 + pBR322_*csgD*_STm_ + pBA | This study |
| JSG5461 | JSG3979 Δ*csgD* JSG4889 + pBR322_*csgD*_STm_ + pDEFG | This study |
| JSG5270 | JSG3979 Δ*csgD* JSG4889 + pWSK29 | This study |
| JSG5268 | JSG3979 Δ*csgD* JSG4889 + pWSK_*csgD*_STm_ | This study |
| JSG3981 | Gallbladder chronic carrier isolate | Gift of S. Baker (Vietnam) |
| JSG4964 | JSG3981 + pBR322 | This study |
| JSG5462 | JSG3981 + pBR322 + pBA | This study |
| JSG5463 | JSG3981 + pBR322 + pDEFG | This study |
| JSG5273 | JSG3981 + pWSK29 | This study |
| JSG4890 | JSG3981 Δ*csgD* | This study |
| JSG4971 | JSG3981 Δ*csgD* JSG4890 + pBR322 | This study |
| JSG5464 | JSG3981 Δ*csgD* JSG4890 + pBR322 + pBA | This study |
| JSG5465 | JSG3981 Δ*csgD* JSG4890 + pBR322 + pDEFG | This study |
| JSG4978 | JSG3981 Δ*csgD* JSG4890 + pBR322_*csgD*_STm_ | This study |
| JSG5466 | JSG3981 Δ*csgD* JSG4890 + pBR322_*csgD*_STm_ + pBA | This study |
| JSG5467 | JSG3981 Δ*csgD* JSG4890 + pBR322_*csgD*_STm_ + pDEFG | This study |
| JSG5278 | JSG3981 Δ*csgD* JSG4890 + pWSK29 | This study |
| JSG5276 | JSG3981 Δ*csgD* JSG4890 + pWSK_*csgD*_STm_ | This study |
| JSG5413 | JSG3981 Δ*csgD* JSG4890 + pWSK_*csgD*_STy_ | This study |
|  |  |  |
| **Plasmid** | **Purpose** | **Source** |
| pBR322 | Empty pBR322 plasmid with Amp and Tet resistance cassette | (Bolivar et al., 1977) |
| pBR_*csgD*_STm_ | pBR322 with a copy of the WT *csgD* gene under the lac promoter for complementation assays; Amp and Tet resistance cassette | This study |
| pWSK29 | Empty pWSK29 plasmid with Amp resistance cassette | (Rong Fu Wang and Kushner, 1991) |
| pWSK_*csgD*_STm_ | pWSK29 with a copy of the WT *csgD* gene under the lac promoter for complementation assays; Amp resistance cassette | This study |
| pWSK_*csgD*_STy_ | pWSK29 with a copy of the WT Typhi *csgD* gene under the lac promoter for complementation assays; Amp resistance cassette | This study |
| pBAC | Reporter vector for Curli *csgBAC* operon in pCS26; Km resistance cassette | (Santander et al., 2007) |
| pDEFG | Reporter vector for Curli *csgDEFG* operon in pCS26; Km resistance cassette | (White et al., 2006) |

References:

1. Bolivar F., Rodriguez R.L., Greene P.J., Betlach M.C., Heyneker H.L., Boyer H.W., Crosa J.H., Falkow S. Construction and characterization of new cloning vehicles. II. A multipurpose cloning system. *Gene.* 1977;2(2):95–113.
2. Rong Fu Wang, F. W., & Kushner, S. R. Construction of versatile low-copy-number vectors for cloning, sequencing and gene expression in Escherichia coli. *Gene*. 1991;100:195–199. doi: 10.1016/0378-1119(91)90366-J.
3. Santander J., Wanda S.-Y., Nickerson C.A., Curtiss R. Role of RpoS in fine-tuning the synthesis of Vi capsular polysaccharide in *Salmonella enterica* serotype Typhi. *Infect. Immun.*2007;75:1382–1392. doi: 10.1128/IAI.00888-06.
4. White AP, Gibson DL, Kim W, Kay WW, Surette MG. Thin aggregative fimbriae and cellulose enhance long-term survival and persistence of *Salmonella*. *J Bacteriol.* 2006;188(9):3219–27. doi: 10.1128/JB.188.9.3219-3227.2006.
