# Supplementary material for: Functional role of the biofilm regulator CsgD in Salmonella enterica sv. Typhi
Source: Front Cell Infect Microbiol. 2024 Dec 11;14:1478488. doi: 10.3389/fcimb.2024.1478488 (PMC11668344; doi:10.3389/fcimb.2024.1478488)
Supplement: Supplementary file 3 [file Table2.docx]

**Supplementary Table 2.** Oligonucleotide primers used in this study

| **Primer** | **Sequence** | **Purpose** |
| --- | --- | --- |
| JG2991 | gtgcgattaaaaaaagtggagtttcatcATGTTTAATGAAGTGTAGGCTGGAGCTGCTTC | Wanner Forward *csgD* |
| JG2992 | aatccaggtcagatagcgtttcatggccTTACCGCCTGAGCATATGAATATCCTCCTTAG | Wanner Reverse *csgD* |
| JG3262 | gaagcataagaacatccccatggcgCACGATCATGCGCACCCGTG | Gibson Assembly Forward 1 for inserting *S.* Typhimurium *csgD* with promoter into pBR322 plasmid |
| JG3263 | CACGGGTGCGCATGATCGTGcgccatggggatgttcttatg | Gibson Assembly Reverse 1 for inserting *S.* Typhimurium *csgD* with promoter into pBR322 plasmid |
| JG3264 | GGTCCTCAACGACAGGAGtcagatagcgtttcatggccTTACCGC | Gibson Assembly Forward 2 for inserting *S.* Typhimurium *csgD* with promoter into pBR322 plasmid |
| JG3265 | GGTAAggccatgaaacgctatctgaCTCCTGTCGTTGAGGACCCG | Gibson Assembly Reverse 2 for inserting *S.* Typhimurium *csgD* with promoter into pBR322 plasmid |
| JG3304 | gagcggataacaatttcacacaggaaacaaagtggagtttcatcatgtttaatgaagtccatag | Gibson assembly Forward 1 for inserting *csgD* gene into pWSK29 plasmid |
| JG3307 | cccactacgtgaaccatcagccttaccgcctgagattatcgtttgcc | Gibson assembly Reverse 1 for inserting *S.* Typhimurium *csgD* gene into pWSK29 plasmid |
| JG3305 | ctactatggacttcattaaacatgatgaaactccactttgtttcctgtgtgaaattgttatccgc | Gibson assembly Forward 2 for inserting *csgD* gene into pWSK29 plasmid |
| JG3306 | gggcaaacgataatctcaggcggtaaggctgatggttcacgtagtgg | Gibson assembly Reverse 2 for inserting *S.* Typhimurium *csgD* gene into pWSK29 plasmid |
| JG3330 | GCACCCAGGCAGTTTCATGAtaaggctgatggttcacgtagtgg | Gibson assembly Reverse 2 for inserting *S.* Typhi *csgD* gene into pWSK29 plasmid |
| JG3331 | ctacgtgaaccatcagccttaTCATGAAACTGCCTGGGTGCG | Gibson assembly Reverse 1 for inserting *S.* Typhi *csgD* gene into pWSK29 plasmid |
